# Pediatric Kidney Transplantation: A Systematic Review

**DOI:** 10.3390/children13081122

**Published:** 2026-08-21

**Authors:** Noor Sadiq Almoosawe, Fatima Majid Alezairej, Sultan Alsalami, Ayesha Mohamed Alkhanbouli, Ghadir Toosi Zadeh, Ahmed Ghazi Saab, Hia Sadiq Almoosawe, Sarah Albadran, Mustafa Alani, Subhranshu Sekhar Kar, Bellary Kuruba Manjunatha Goud, Rajani Dube

**Affiliations:** RAK College of Medical Sciences, Ras Al Khaimah Medical and Health Sciences University, Ras Al Khaimah P.O. Box 11172, United Arab Emirates; fatimah.23901020@rakmhsu.ac.ae (F.M.A.); sultan.23901089@rakmhsu.ac.ae (S.A.); ayesha.23901012@rakmhsu.ac.ae (A.M.A.); ghadir.23901023@rakmhsu.ac.ae (G.T.Z.); ahmed.23901007@rakmhsu.ac.ae (A.G.S.); hia.25901106@rakmhsu.ac.ae (H.S.A.); sarah.23901081@rakmhsu.ac.ae (S.A.); mustafa.23901060@rakmhsu.ac.ae (M.A.); manjunatha@rakmhsu.ac.ae (B.K.M.G.); rajani.dube@rakmhsu.ac.ae (R.D.)

**Keywords:** kidney transplantation, pediatric, systematic review, graft survival, end-stage renal disease

## Abstract

**Highlights:**

**What are the main findings?**

- This review synthesizes the short- and long-term patient and graft survival rates in pediatric kidney transplantation, establishing it as the optimal intervention for end-stage renal disease.
- The analysis maps a multi-dimensional spectrum of post-transplant outcomes, pinpointing critical clinical, surgical, and immunological determinants that accelerate graft dysfunction.

**What is the implication of the main finding?**

- Identifying specific transplant-related risk factors provides clinicians with actionable insights to anticipate and mitigate early and late-stage graft failure.
- These findings help bridge current literature gaps, guiding the optimization of surgical strategies and tailored post-operative protocols to improve long-term pediatric graft longevity.

**Abstract:**

Background/Objectives: Kidney transplantation is the most effective treatment for pediatric patients with end-stage renal disease (ESRD), offering superior survival rates and quality of life compared to dialysis. However, long-term graft survival remains a complex clinical and surgical challenge. This systematic review aims to evaluate short- and long-term graft outcomes and comprehensively analyze the primary clinical, immunological, and procedural risk factors driving pediatric graft failure. Methods: A comprehensive search was conducted across the PubMed database, yielding 289 initial records. Following duplicate removal and systematic title and abstract screening, 108 full-text articles were evaluated for eligibility. Ultimately, 35 high-quality studies met the full inclusion criteria and were selected for final data synthesis and analysis. Results: Synthesized data revealed high short-term outcomes, with a 1-year graft survival rate of 94.60%. However, survival experienced a steady decline over time, dropping to 59.50% at the 10-year mark. Acute rejection episodes and delayed graft function (DGF) were identified as the primary immunological primary risk factors. Furthermore, specific donor characteristics, early surgical complications (such as vascular thrombosis), post-transplant infections affecting nearly 45% of patients, and adolescent non-adherence to immunosuppressive regimens were heavily associated with accelerated graft failure. Conclusions: While short-term success in pediatric kidney transplantation is highly encouraging, long-term graft longevity requires targeted clinical interventions. Optimizing outcomes necessitates precise, lifelong management strategies specifically focused on mitigating chronic rejection, preventing post-operative infections, and implementing multidisciplinary support systems to enhance patient adherence to immunosuppressive therapy.

## 1. Introduction

Kidney transplantation is widely recognized as the definitive and most effective therapeutic strategy for children suffering from end-stage renal disease (ESRD) [1,2]. Compared with long-term dialysis, successful kidney transplantation provides significantly better patient survival, while markedly improving growth, pubertal development, psychosocial well-being, educational achievement, and overall quality of life [1]. Recent long-term cohort studies have further confirmed these benefits, demonstrating excellent patient survival, sustained graft function, improved physical development, and favorable long-term social outcomes among pediatric kidney transplant recipients, particularly when optimal graft function is maintained and transplantation is performed early in childhood [3]. Over the past several decades, continuous advances in microsurgical techniques, donor selection, individualized immunosuppressive regimens, and comprehensive postoperative monitoring have substantially improved early graft and patient survival, making pediatric kidney transplantation the standard of care for children with ESRD [4,5].

**Figure 1:**
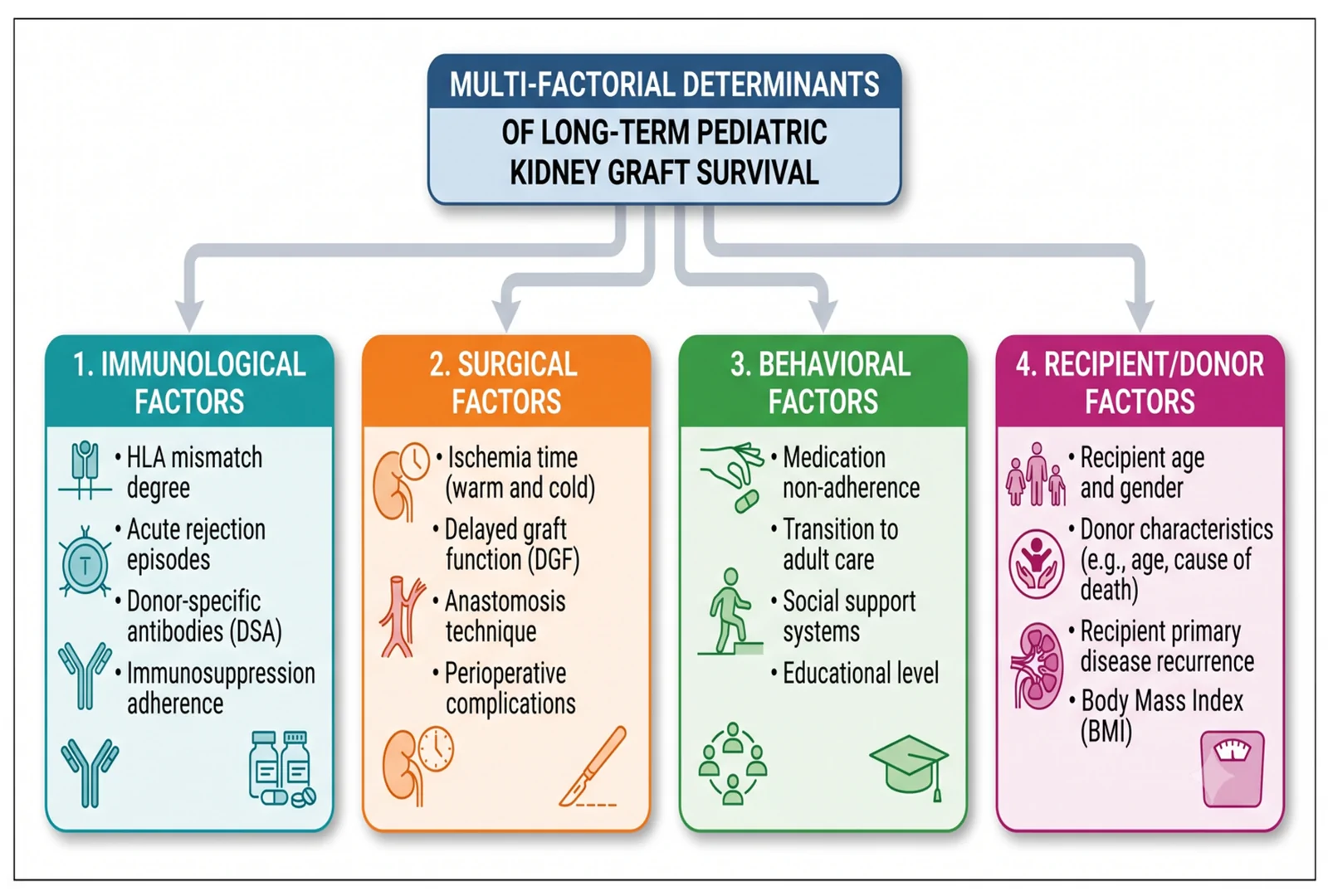
Schematic diagram of long-term graft survival determinants.

Despite these remarkable advances, pediatric kidney transplantation remains a highly complex clinical challenge because long-term graft survival is influenced by numerous immunological, surgical, behavioral, and recipient-related factors (Figure 1) [6,7]. Figure 1. Schematic representation of multi-factorial determinants influencing long-term pediatric kidney graft survival, categorized into immunological, surgical, behavioral, and recipient-related domains. Children generally exhibit a more vigorous immune response than adults, increasing their susceptibility to acute rejection and chronic graft injury [8,9]. Recent large-scale analyses have identified recipient age, donor age, obesity, and a high degree of human leukocyte antigen (HLA) mismatch as independent predictors of acute rejection, highlighting the need for individualized immunosuppressive strategies and careful donor–recipient matching to improve long-term graft outcomes [8]. Likewise, delayed graft function has recently been shown to independently predict inferior graft and patient survival, even after adjustment for multiple donor and recipient risk factors, emphasizing the importance of minimizing perioperative injury and optimizing early graft performance [10]. Furthermore, recent evidence suggests that although preemptive kidney transplantation is increasingly performed at higher estimated glomerular filtration rate (eGFR) levels, earlier transplantation alone does not necessarily improve long-term graft or patient survival, indicating that multiple clinical variables beyond transplant timing determine long-term outcomes [11].

Long-term transplant success also depends heavily on standard lifelong follow-up after surgery. Pediatric recipients remain vulnerable to infectious complications, medication nonadherence, metabolic disorders, hypertension, donor-specific antibodies, recurrent primary kidney disease, and chronic allograft dysfunction, all of which contribute to progressive graft deterioration over time [6,12]. Recent reports have demonstrated that acute graft rejection continues to represent one of the greatest threats to graft survival, with donor-specific antibodies and HLA-DR mismatching serving as major independent predictors of rejection, while viral infections such as Epstein–Barr virus (EBV), cytomegalovirus (CMV), and BK virus remain frequent post-transplant complications requiring continuous surveillance [13]. Moreover, adolescence represents a particularly vulnerable period because medication nonadherence during the transition from pediatric to adult nephrology services substantially increases the risk of graft rejection and graft loss. Contemporary evidence emphasizes that structured transition programs, multidisciplinary care, adherence monitoring, and patient education are essential to improve long-term outcomes during this critical phase [14,15].

Although numerous studies have investigated pediatric kidney transplantation, substantial variability remains across transplant centers regarding patient survival, graft longevity, and the clinical factors associated with transplant success [16,17]. These differences likely reflect variations in donor characteristics, recipient demographics, immunosuppressive protocols, perioperative management, and long-term follow-up practices [18,19]. Recent multicenter cohort studies further confirm that pediatric kidney transplant outcomes are determined by a complex interaction of immunological, surgical, behavioral, and clinical factors, rather than by a single determinant, reinforcing the importance of individualized patient management throughout childhood and adolescence [20].

Consequently, this systematic review was designed to synthesize the available global evidence regarding pediatric kidney transplantation outcomes. By integrating findings from recent cohort studies, registry analyses, and contemporary clinical investigations, this review aims to identify the principal immunological, surgical, recipient-related, and behavioral determinants of graft survival while providing a comprehensive overview of the factors that influence long-term patient and graft outcomes. Ultimately, this review seeks to support evidence-based strategies that optimize transplant care, prolong graft longevity, and improve the lifelong health and quality of life of pediatric kidney transplant recipients. While previous reviews have broadly addressed pediatric renal transplantation, this systematic review provides an updated, multi-dimensional synthesis specifically focusing on contemporary 2010–2025 cohort outcomes, long-term multi-factorial graft failure determinants, and the critical clinical-behavioral transition challenges faced by adolescent recipients.

## 2. Methods

A comprehensive and systematic literature search was conducted using the PubMed database to identify relevant studies evaluating clinical outcomes in pediatric kidney transplantation. The search strategy was restricted to articles published between 2010 and 2025, utilizing targeted keywords and Medical Subject Headings (MeSH) terms related to “pediatric kidney transplantation,” “graft survival,” “patient survival,” and “transplant outcomes” to ensure a thorough retrieval of potentially eligible literature.

This systematic review was conducted in strict accordance with the Preferred Reporting Items for Systematic Reviews and Meta-Analyses (PRISMA) 2020 guidelines. The review protocol was prospectively registered with the International Prospective Register of Systematic Reviews (PROSPERO) under the registration number CRD420261439976.

**Figure 2:**
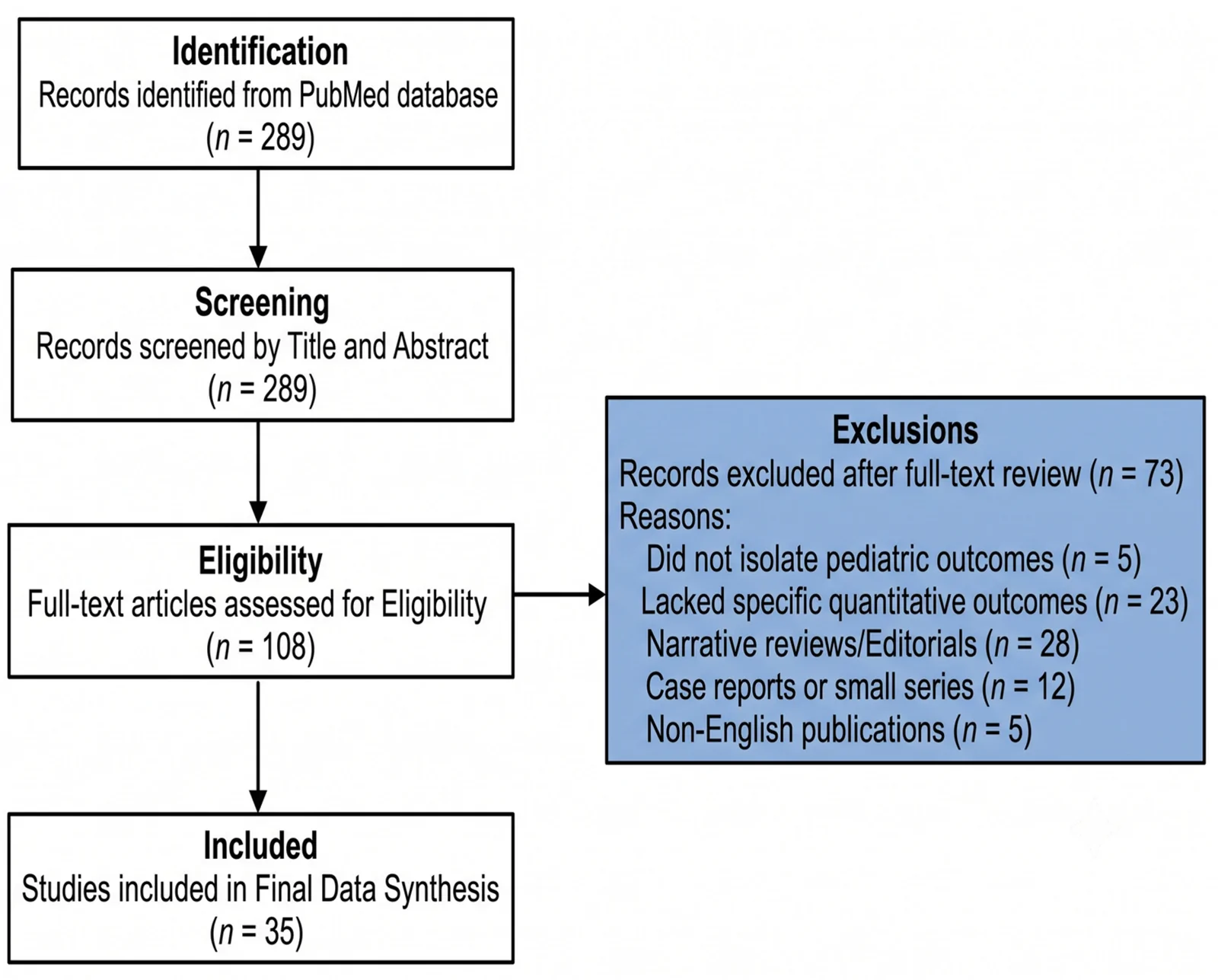
PRISMA 2020 flow diagram of the study identification, screening, and selection process.

The selection process followed a systematic, multi-stage screening approach. Initially, the titles and abstracts of all captured records were independently screened to filter out duplicates and assess their broad relevance to our core research questions. Studies meeting our foundational eligibility criteria advanced to the next stage. Subsequently, a systematic full-text review was performed on the remaining articles to definitively judge their suitability, focusing on study design, patient population, and the clarity of reported graft outcomes. Ultimately, only high-quality studies that strictly satisfied our predefined inclusion and exclusion criteria were preserved for final data synthesis. The entire study selection and screening process is comprehensively detailed in the accompanying PRISMA flow diagram (Figure 2).

### 2.1. Search Strategy

To identify potentially relevant literature, we executed a systematic search across the PubMed database using targeted combinations of medical terminology. The core keywords included: “pediatric kidney transplantation,” “renal transplantation in children,” “graft survival,” “patient survival,” and “transplant outcomes.” To refine the search and capture the most comprehensive set of records, these terms were structurally combined using the Boolean operators AND and OR, (e.g., “pediatric kidney transplantation” OR “renal transplantation in children” AND “graft survival”).

This approach was tailored specifically to isolate clinical trials, cohort studies, and observational data focusing on pediatric transplant recipients. The resulting records were then standard cataloged and moved to the multi-stage screening process to align directly with the primary objectives of this systematic review.

### 2.2. Inclusion Criteria

Studies were selected and deemed eligible for final analysis if they strictly fulfilled the following criteria:

- Target population: Studies explicitly involving pediatric patients under the age of 18 who had undergone kidney transplantation.
- Outcome measures: Studies reporting definitive clinical endpoints, specifically including graft survival rates, patient survival rates, or immediate post-transplant complication outcomes.
- Temporal frame: Original research articles published within a 15-year window between January 2010 and December 2025, capturing modern immunosuppressive and surgical eras.
- Language and accessibility: Peer-reviewed studies fully available in the English language, with accessible abstract and full-text formats.

### 2.3. Exclusion Criteria

Studies were excluded from this systematic review if they met any of the following non-eligibility conditions:

- Ineligible populations: Research focusing exclusively on adult transplant recipients, or mixed-cohort studies where pediatric data could not be independently extracted.
- Methodological and publication types: Secondary literature, including review articles, systematic reviews, meta-analyses, and editorials, as well as descriptive single-patient case reports or very limited case series.
- Linguistic barriers: Studies where the full text was not published or available in the English language.
- Irrelevant outcomes: Studies that completely lack definitive data on clinical transplant endpoints, specifically failing to report on graft survival, patient survival, or post-operative functional outcomes.

### 2.4. Study Selection

The initial search of the PubMed database identified 289 records related to pediatric kidney transplantation outcomes. After removing 36 duplicate records, 253 studies remained for title and abstract screening. During this stage, 145 articles were excluded because they did not meet the predefined inclusion criteria or were not relevant to the research question. A total of 108 full-text articles were then assessed for eligibility. Each study was carefully reviewed to determine whether it met the inclusion criteria for this systematic review. After the full-text assessment, 73 articles were excluded for the following reasons: studies involving only adult populations (n=5), absence of specific quantitative transplant outcome data (n=23), narrative reviews, editorials, or opinions (n=28), case reports or very small case series (n=12), and non-English publications (n=5). Finally, 35 high-quality studies met all inclusion criteria and were included in this systematic review as detailed in the PRISMA flow diagram (Figure 2). The methodological quality of the included cohort studies is summarized in Table 1, while the synthesized clinical outcomes and complication rates are presented in Table 2.

The pooled estimates demonstrate excellent short-term graft survival, with a 1-year survival rate of 94.60% (95% CI: 92.8–96.2%). Although medium-term outcomes remain stable at 5 years (88.20%), a significant long-term decline is observed by year 10, where graft survival drops to 59.50% (95% CI: 55.0–64.0%). In terms of post-operative complications, infections were the most common non-immunological event, affecting approximately 45% of patients. Immunological and functional challenges were also prominent, with acute rejection and delayed graft function (DGF) reported at pooled estimates of 25% and 20%, respectively. Conversely, vascular complications were less frequent (6.8%) but represent a critical risk factor for early graft loss.

### 2.5. Characteristics of Included Studies

The 35 studies compiled in this systematic review spanned a diverse geographical range, reflecting global practices and outcomes in pediatric kidney transplantation. The patient cohorts and sample sizes varied substantially across the literature—ranging from focused, single-center retrospective reviews to massive, multi-center registry analyses. Despite this structural diversity, the vast majority of these studies consistently tracked core clinical endpoints, centering primarily on chronological graft survival, patient survival rates, and the incidence of post-transplant complications.

### 2.6. Summary of Graft Survival Findings

A cross-examination of the gathered evidence reveals a highly encouraging trend in early post-transplant outcomes. Overall, the literature points to high short-term graft survival, with the highest success rates concentrated within the immediate years following surgery [16,26]. The data demonstrate that when modern, standard surgical techniques are paired with proactive, standard postoperative monitoring, early graft outcomes are overwhelmingly positive [25,36].

However, maintaining this success over the long term remains a far more volatile challenge, heavily dictated by an intricate mix of clinical variables. Both delayed graft function (DGF) and early acute rejection episodes were universally flagged across the studies as the most aggressive and reliable predictors of premature graft loss [10,23,30,42,43,44]. Furthermore, donor-specific variables introduce another layer of complexity; the type of donor (living versus deceased) and extreme donor age caps (very young or older donors) were shown to significantly sway the trajectory of graft longevity [18,45,46].

Recipient-specific characteristics and behaviors also heavily shape long-term survival curves. Several long-term follow-up studies noted a troubling trend: older adolescents and young adults exhibit notably higher rates of graft failure compared to their younger pediatric counterparts [6,47]. This vulnerability in older pediatric cohorts is frequently driven by behavioral hurdles—most notably a documented drop in adherence to daily immunosuppressive regimens [6,12]. Additionally, non-immunological complications, with post-transplant bacterial and viral infections leading the charge, were heavily implicated in triggering chronic graft dysfunction and subsequent failure [34,48,49].

In summary, while the contemporary literature confirms that pediatric kidney transplantation yields outstanding immediate and short-term graft survival, securing these organs for the future is highly dependent on mitigating donor–recipient mismatches, managing early functional delays, and supporting young patients through their long-term therapeutic journeys (Table 3).

### 2.7. Risk Factors for Graft Failure

Synthesizing the data across the included studies underscores that pediatric graft survival is rarely dictated by a single variable. Instead, graft failure is driven by a multifaceted interplay of clinical hurdles. These primary risk factors can be broadly organized into five critical domains: immunological challenges, donor-specific characteristics, early post-transplant graft function, clinical or surgical complications, and behavioral factors (Table 4).

### 2.8. Immunologic Factors

Immunological complications remain among the most formidable barriers to sustained graft survival in children. A major challenge post-transplantation is the recurrence of primary glomerular diseases. Specifically, focal segmental glomerulosclerosis (FSGS)—which represents a heterogeneous histological pattern of injury resulting from diverse primary immunologic, genetic, or circulating podocyte-toxic mechanisms—was consistently identified as a prominent risk factor for post-transplant recurrence and premature graft failure [21,54]. Alongside disease recurrence, acute rejection episodes stand out as powerful, direct predictors of abbreviated long-term graft survival [8,39,42]. The literature emphasizes that a single past episode of acute rejection can set off a domino effect, leading to progressive chronic rejection, accelerated tissue injury, and eventual allograft failure if not aggressively managed with optimized induction protocols, secondary immunosuppression, and steroid-sparing strategies [55,56,57,58,59,60]. Furthermore, specialized immunologic barriers such as ABO-incompatible transplantation require rigorous desensitization and immunoadsorption protocols to prevent acute humoral injury [61].

### 2.9. Donor-Related Factors

The structural and genetic baseline of the donated organ heavily dictates how well it adapts to a young recipient. Key donor-related determinants influencing survival outcomes include extreme donor age (with very young deceased donors and acute kidney injury donors presenting unique technical challenges), deceased versus living donor status, and human leukocyte antigen (HLA) mismatching [19,45,62,63]. In contrast, preemptive transplantation strategies—particularly in very young pediatric cohorts—yield substantial graft and patient survival advantages [1,64]. Technical innovations, such as en-bloc transplantation from very small pediatric deceased donors, have also demonstrated viable long-term graft longevity despite increased surgical complexity [46,65]. Furthermore, structural donor–recipient size mismatches—such as a low donor-to-recipient body weight ratio—were shown to compromise long-term graft hemodynamics [52,66,67]. Interestingly, some studies noted that broader macro-level variables, including institutional organ allocation policies and distribution systems, also indirectly shape pediatric transplant success rates by altering ischemic times [68,69].

### 2.10. Early Post-Transplant Graft Function

The immediate performance of the kidney in the operating room and early post-operative days serves as a critical indicator for its long-term survival. Both delayed graft function (DGF) and slow graft function are heavily implicated in poorer long-term outcomes and a heightened risk of graft loss [23,43,70]. These early functional delays typically mirror underlying ischemia–reperfusion injuries, perioperative complications, and non-immunologic allograft injury that blunt the organ’s initial performance, leaving it more vulnerable to subsequent graft failure [10,33,71].

### 2.11. Clinical and Surgical Complications

Beyond immunity, a child’s graft must withstand various anatomical and systemic stressors. Systemic clinical conditions such as chronic post-transplant hypertension and medication-induced nephrotoxicity (often from calcineurin inhibitors) can trigger a slow, progressive decline in graft function over time [6,72,73]. On the surgical front, early vascular complications—most notably renal vein thrombosis and renal artery stenosis—are acute, catastrophic events that frequently culminate in immediate graft loss if surgical intervention fails [25,36,37]. Additionally, early intra-abdominal surgical complications and post-operative urological anomalies (such as ureteral obstructions) present significant structural threats to early graft integrity [24,38,74].

### 2.12. Recipient-Related and Behavioral Factors

The intrinsic characteristics and developmental stages of the young recipient significantly impact the life of the allograft. A distinct and troubling trend highlighted across the literature is that graft failure rates climb sharply as children transition into adolescence and early adulthood [6,47]. This specific developmental window introduces immense psychological and behavioral hurdles, most notably a documented drop in adherence to daily medication routines [14,75]. Consequently, clinical non-adherence to immunosuppressive regimens emerged as one of the most critical behavioral drivers behind late acute rejection, progressive graft dysfunction, and preventable organ loss in this vulnerable age group [6,12] (Table 5).

### 2.13. Infection-Related Factors

Post-transplant infectious complications act as a continuous double-edged sword, threatening the graft both through direct tissue damage and by triggering host alloimmunity. Chronic viral infections, particularly BK polyomavirus and cytomegalovirus (CMV), are frequently encountered in pediatric recipients and are heavily implicated in driving nephropathy and subsequent graft dysfunction [35,78,79]. Systemic respiratory and viral outbreaks, including COVID-19 infection in pediatric transplant recipients, have also posed notable clinical risks requiring temporary adjustments in immunosuppression [80]. Furthermore, the onset of opportunistic bacterial or fungal infections during intense periods of immunosuppression not only results in high hospitalization rates but also strongly correlates with subsequent rejection episodes and compromised long-term transplant outcomes [34,48,49].

### 2.14. Other Clinical Predictors

Beyond mainstream immunological and surgical variables, several secondary clinical baselines further refine long-term survival predictions. Longitudinal trajectories of allograft function provide essential insights into progressive chronic damage [81]. Recipient obesity and pre-existing metabolic syndromes create unfavorable hemodynamics that accelerate graft aging [39,40]. Similarly, children undergoing retransplantation face vastly superior immunological and technical hurdles compared to primary transplant recipients [82]. Structural abnormalities, such as pre-existing urological anomalies or severe donor–recipient size mismatches, also act as persistent threats that blunt both immediate functional recovery and extended allograft survival [24,52,67]. Composite prognostic indicators and time-varying determinants are increasingly utilized to map long-term graft failure trajectories across diverse cohorts [83,84].

## 3. Post-Transplant Complications and Patient Outcomes

Evaluating post-transplant complications is essential to understanding the broader picture of patient survival and long-term allograft vitality. The clinical journey of a pediatric transplant recipient is often complicated by a shifting landscape of metabolic disorders, immunological events, chronic infections, malignancies, and cardiovascular stressors.

### 3.1. Metabolic Complications

Metabolic instability is a frequent consequence of long-term immunosuppression and altered renal physiology. Chronic hyperuricemia stands out as a prevalent metabolic complication in young recipients, demonstrating a strong correlation with a reduced estimated glomerular filtration rate (eGFR) and a progressive decline in graft function over time [40]. Similarly, the onset of post-transplant diabetes mellitus (PTDM) and severe carbohydrate intolerance compromises metabolic homeostasis, creating a toxic environment that significantly elevates the risk of graft dysfunction and aggressive rejection episodes [41].

### 3.2. Immunologic Complications

Immunological vigilance remains critical long after the immediate post-operative phase. Beyond classic acute cellular rejection, the insidious development of de novo donor-specific antibodies (DSAs) and subsequent antibody-mediated rejection (AMR) present persistent threats to tissue integrity [57,58]. Surveillance biopsies have also exposed subclinical inflammation phenotypes, which act as early, silent precursors to chronic graft deterioration [85]. Furthermore, recent insights highlight that non-HLA antibodies—specifically those targeting G protein-coupled receptors—act as powerful alternative immune mechanisms that trigger vascular injury and accelerate graft loss [86,87].

### 3.3. Infectious Complications

Maintaining a mandatory therapeutic balance of immunosuppression inherently leaves pediatric patients vulnerable to life-threatening pathogens. Viral reactivation is highly common, with Epstein–Barr virus (EBV), cytomegalovirus (CMV), BK polyomavirus (BKPyV), and severe gastrointestinal viral pathogens such as norovirus driving a significant proportion of post-transplant morbidity and chronic dysfunction [35,78,88,89]. Managing these viral challenges primarily hinges on the judicious reduction or modification of baseline immunosuppressive regimens to enable host clearance while avoiding acute rejection. Target-specific pharmacotherapies are crucial: intravenous ganciclovir or oral valganciclovir serves as the cornerstone for CMV prophylaxis and preemptive therapy, whereas BKPyV nephropathy requires step-down immunosuppression, occasionally augmented by adjuvant agents like IVIG or cidofovir in severe refractory cases. For opportunistic bacterial and fungal pathogens, empirical or culture-guided systemic antimicrobial therapy (such as trimethoprim-sulfamethoxazole for Pneumocystis jirovecii prophylaxis and fluconazole or echinocandins for severe fungal infections) combined with close therapeutic drug monitoring (TDM) is routinely executed to prevent graft-threatening systemic toxicity.

### 3.4. Malignancies

Though oncological events remain statistically less frequent in children than in adults, the cumulative impact of long-term immunosuppressive regimens means pediatric kidney transplant recipients face a significantly higher cancer risk compared to the general population [90,91,92]. Pre-transplant malignancy history and post-transplant de novo oncogenesis require rigorous lifelong oncological surveillance. Post-transplant lymphoproliferative disorder (PTLD) is the most prominent and frequently documented malignancy in this cohort, heavily driven by primary or reactivated EBV infections under intense immune suppression [93,94,95]. Therapeutic strategies for PTLD primarily begin with the prompt reduction or withdrawal of baseline immunosuppression to allow host T-cell immune reconstitution. In cases of CD20-positive B-cell PTLD, targeted immunotherapy using the anti-CD20 monoclonal antibody rituximab serves as first-line treatment, often combined with low-dose chemotherapy regimens (e.g., CHOP-like protocols) or adoptive EBV-specific cytotoxic T-cell therapy for refractory or aggressive lesions.

### 3.5. Cardiovascular Risk Factors and Patient Survival

Systemic cardiovascular stressors heavily influence the lifespan of both the patient and the organ. Chronic post-transplant hypertension is highly prevalent in pediatric cohorts, serving as a silent driver of vascular remodeling, cardiovascular complications, and chronic allograft nephropathy [6,73].

Despite this daunting array of potential complications, contemporary literature delivers an overwhelmingly reassuring message regarding overall patient survival. Driven by decades of iterative refinements in fine-scale surgical techniques, steroid-avoidance or minimization protocols, and proactive multidisciplinary care, 1-year patient survival rates consistently exceed 95% [12,31,96,97]. Ultimately, while pediatric kidney transplantation offers an unparalleled bridge to superior survival and a restored quality of life, maximizing these benefits requires lifelong, standard surveillance to intercept complications early and protect the child’s long-term health [4,6,12] (Table 6).

## 4. Discussion

This systematic review synthesizes current global evidence evaluating clinical outcomes in pediatric kidney transplantation and uncovers the critical drivers behind allograft failure. Broadly speaking, the compiled literature confirms that pediatric kidney transplantation delivers outstanding short-term success, boasting high graft and patient survival rates throughout the immediate post-operative years. However, maintaining this initial success over an extended timeline remains a significant clinical challenge, as long-term graft survival trajectories vary significantly and exhibit a steady, longitudinal decline over time.

A deeper dive into these survival curves reveals that graft failure is rarely driven by an isolated event, but rather by an overlapping network of clinical and behavioral stressors. Immunological hurdles remain among the most aggressive barriers to graft longevity. Both acute rejection episodes and the post-transplant recurrence of the patient’s primary renal pathology were universally flagged as powerful predictors of premature organ loss. Specifically, diseases with a notorious propensity for recurrence, such as focal segmental glomerulosclerosis (FSGS), present a persistent clinical threat, frequently triggering aggressive allograft dysfunction shortly after surgery.

Beyond baseline immunity, the immediate performance of the newly implanted kidney functions serves as a critical predictor for its future viability. Suboptimal early graft function—manifesting either slow graft function or overt delayed graft function (DGF)—consistently correlates with inferior long-term survival curves and a heightened vulnerability to subsequent failure. These findings emphasize that mitigating perioperative ischemic injuries and securing strong initial graft perfusion are paramount to shielding the organ from early functional blunting.

Donor-specific profiles and macro-level allocation strategies also exert a profound structural influence on transplant outcomes. Baseline variables such as donor age, donor type, human leukocyte antigen (HLA) matching, and institutional organ distribution policies all significantly shape the graft’s lifespan. Reassuringly, the literature heavily demonstrates that living donor transplantation and preemptive transplantation strategies grant children a substantial survival advantage, yielding vastly superior outcomes compared to undergoing transplantation after enduring years of debilitating chronic dialysis.

In addition to genetic and surgical variables, the long-term health of the graft is inextricably linked to behavioral patterns and systemic complications. The transition through adolescence and early adulthood introduces immense psychological friction, and medical non-adherence to daily immunosuppressive regimens stands out as a leading behavioral driver of preventable chronic rejection and late graft failure in these young patients. Furthermore, non-immunological complications—such as chronic post-transplant hypertension, structural or vascular surgical anomalies, metabolic disorders like post-transplant diabetes, and opportunistic bacterial or viral infections—introduce a continuous barrage of stressors that can accelerate allograft degradation.

Despite the daunting reality of these multi-layered complications, contemporary data delivers an overwhelmingly encouraging narrative. Driven by decades of iterative refinements in fine-scale micro-surgical techniques, steroid-minimization protocols, and sophisticated post-transplant monitoring, early patient and graft survival metrics have reached historic highs.

Ultimately, kidney transplantation remains the undisputed gold standard of care for children battling end-stage renal disease (ESRD), fundamentally restoring both their physical health and overall quality of life. Moving forward, securing these organs for the future will depend heavily on our ability to optimize donor–recipient matching, refine targeted immunosuppression, proactively curb infectious risks, and provide comprehensive, multidisciplinary support to help young patients navigate their lifelong therapeutic journeys.

## 5. Conclusions

Pediatric kidney transplantation remains the absolute gold standard of care for children battling end-stage renal disease (ESRD), offering an unparalleled bridge to superior survival rates and a fundamentally restored quality of life. However, securing these life-changing benefits over an extended timeline is a continuous challenge, as long-term graft survival remains highly sensitive to a complex network of clinical and behavioral variables. Longevity is ultimately decided by how effectively clinicians can navigate immunological hurdles, mitigate donor–recipient mismatches, prevent early post-operative functional delays, and manage critical patient-centered factors such as treatment adherence and infectious risks. Moving forward, a commitment to systematic, lifelong monitoring and a proactive, multidisciplinary approach to managing these interlocking risk factors is paramount to extending allograft survival and safeguarding the long-term health of these young recipients.

## Figures and Tables

**Table 1:** Illustrative Summary of JBI Quality Appraisal for Selected Cohort Studies.

Overall Appraisal	Sample Size	Data Source	Study Design	Author (Year)
High quality	3010	NAPRTCS registry	Retrospective cohort	Koh et al. (2019) [21]
High quality	7785	UNOS database	Registry cohort	Anand et al. (2021) [22]
High quality	6480	SRTR database	Registry cohort	Hwang et al. (2023) [23]

Note: Quality appraisal was conducted using the Joanna Briggs Institute (JBI) Critical Appraisal Checklist for Cohort Studies.

**Table 2:** Summarizes the synthesized clinical outcomes and complication rates.

Outcome Domain	No. of Studies	References	Total Sample Size (*n*)	Range (%)	Pooled Estimate	95% CI (Approx.)	Interpretation
1-Year graft survival	4	[16,24,25,26]	736	91–97%	94.60%	92.8–96.2%	Excellent short-term survival
5-Year graft survival	5	[16,24,25,26,27]	900	84–93%	88.20%	85.5–90.5%	Stable medium-term outcomes
10-Year graft survival	3	[25,28,29]	571	53–65%	59.50%	55.0–64.0%	Significant long-term decline
Delayed graft function (DGF)	3	[10,23,30]	1200+	10–30%	20%	17–23%	Strong predictor of graft loss
Acute rejection	3	[21,31,32]	800+	10–40%	25%	20–30%	Major immunologic factor
Infection rates	3	[33,34,35]	600+	20–70%	45%	35–55%	Common complication
Vascular complications	3	[36,37,38]	500+	5–8.5%	6.8%	5–8%	Early graft loss risk
Metabolic complications	3	[39,40,41]	700+	10–30%	20%	15–25%	Long-term dysfunction contributor

**Table 3:** Graft Survival Outcomes in Pediatric Kidney Transplantation.

Author and Year [Reference Number]	Country	Study Design	Data Source/Setting	Sample Size	Graft Survival Outcome
Hwang et al., 2023 [23]	United States	Retrospective cohort study	Scientific registry of transplant recipients (SRTR) database	6480 pediatric living donor kidney transplant recipients	Delayed graft function was associated with poorer graft survival compared with transplants without delayed graft function
Amaral et al., 2016 [1]	United States	Retrospective cohort study	USRDS registry data	7527 pediatric kidney transplant recipients	Pre-emptive transplantation improved graft survival compared with dialysis first.
Beetz et al., 2021 [25]	Germany	Retrospective cohort study	Medical and surgical records review	221 pediatric kidney transplant recipients	Graft survival was 97.1% (1 year), 88.9% (5 years), 65.1% (10 years).
Nehus et al., 2017 [31]	United States	Retrospective cohort study	Organ Procurement and Transplantation Network (OPTN) database	3248 pediatric kidney transplant recipients	Five-year graft survival was slightly higher with steroid avoidance therapy (84.8% vs. 81.2%).
Wasik et al., 2019 [18]	United States	Retrospective cohort study	Scientific Registry of Transplant Recipients (SRTR)/(OPTN) database	7155 pediatric kidney transplant recipients	Donor characteristics summarized by the pediatric living kidney donor profile index (P-LKDPI), including factors such as donor age, HLA mismatch, and donor kidney function, were associated with graft survival.
White et al., 2024 [46]	United States	Retrospective cohort study	National transplant registry data (OPTN/ SRTR)	25,486 kidney transplants (225 en-bloc)	One-year graft survival was 96% for larger pediatric donors and 80% for the smallest donors.
Pérez- Bertólez et al., 2017 [50]	Spain	Retrospective single-center cohort study	Single-center chart review	91 pediatric kidney transplant recipients	Living donor graft survival was 96.7% vs. 88% in deceased donor transplantation.

**Table 4:** Risk Factors for Graft Failure in Pediatric Kidney Transplantation.

Author and Year [Reference Number]	Country/Region	Study Design	Data Source/Setting	Sample Size	Key Findings
Koh L.J. et al., (2019) [21]	North America	Retrospective registry cohort (2002–2016)	Registry-based data (NAPRTCS)	3010 total; 455 pediatric recipients with FSGS	5-year graft survival for FSGS was 74.3% vs. 87.1% for other glomerular diseases. Delayed graft function (DGF) was the strongest independent predictor of graft loss (HR 4.39). Living donors did not confer a survival advantage. Immunosuppression type was not associated with outcome.
Anand A. et al., (2021) [22]	USA	Retrospective registry cohort (UNOS database, 1998–2008)	Registry-based data from solid organ transplant database (UNOS)	7785 pediatric kidney transplant recipients	Long-term (10-year) graft survival varied by recipient age; older pediatric age groups had progressively worse graft survival. While acute rejection per se was not the primary focus in this abstract, demographic factors—including age and ethnicity—were identified as significant risk factors for decreased long-term graft survival.
Kaboré R. et al., [51]	France	Retrospective cohort with dynamic prediction modeling (transplants 2002–2013)	Registry-based data (REIN registry)	793 pediatric kidney transplant recipients	Dynamic prediction models that incorporated post-transplant eGFR trajectory (longitudinal graft function) strongly improved prediction of future graft failure compared with baseline predictors alone (joint models AUC 0.83–0.91 vs. Cox model 0.56–0.64). Accounting for longitudinal graft function markedly increased accuracy in identifying patients at risk of graft failure.
Gorchs R. et al., (2025) [8]	USA	Retrospective registry cohort (UNOS data, 2005–2022)	Registry-based data (UNOS)	10,126 pediatric kidney transplant recipients	Identified significant risk factors for acute rejection in first year post-transplant, including younger donor age ≤10 years, high BMI, high HLA mismatch, and recipient age 15–18 years. Protective factors included male sex and recipient 5–10 years. Acute rejection remains an important event that clinicians should address to optimize immunosuppression.
Kostakis I.D. et al., (2023) [52]	USA	Retrospective UNOS registry cohort	Registry database (UNOS)	14,322 pediatric kidney transplants	Donor–recipient size mismatch did not significantly affect delayed graft function (DGF) or primary non-function rates. However, graft survival was significantly higher when donor size relative to recipient body surface area (BSAi) was >2, suggesting a protective effect of larger donor size on long-term survival.
Trnka P. et al., (2018) [19]	Australia/New Zealand	Retrospective registry cohort (ANZDATA, 1990–2015)	Registrybased data	1134 pediatric kidney transplant recipients	Higher donor/recipient age difference and greater HLA mismatch were significantly associated with decreased long-term graft survival. Graft survival at 1, 5, 10, and 15 years was 93.8%, 82.5%, 65.8%, and 49.9%, respectively, with worse outcomes for deceased donors compared with living donors. Increasing donor/recipient age gap (aHR per 10 yr) and each additional HLA mismatch independently predicted graft loss.
Hogan J. et al., (2023) [53]	Not specified	Retrospective international observational cohort (20 centers)	Registry/clinical biopsy and lab data	706 pediatric kidney transplant recipients evaluated	Validated the iBox predictive system for long-term graft failure in children. The model predicted outcomes up to 10 years with strong discrimination (Cindex 0.81) and good calibration across centers. The iBox performed similarly in pediatric recipients as in adult cohorts, supporting its use for risk stratification and monitoring

**Table 5:** Summary of Recipient Characteristics and Adherence-Related Risk Factors in Pediatric Kidney Transplantation.

Author and Year [Reference #]	Country	Study Design	Data Source/Setting	Sample Size	Key Findings
Lentine et al., 2025 [69]	USA	National report	Data analysis	28,142	Five-year graft survival ranged from 86% to 93% depending on donor type.
Nunez et al., 2025 [76]	USA	Retrospective cohort	Medical records	283	Immigration status did not significantly affect graft survival.
Aoki et al., 2022 [77]	Japan	Retrospective cohort	Clinical follow up	375	Post-transplant malignancy occurred in 5.6% of recipients.

**Table 6:** Post-transplant complications and patient outcomes.

Author & Year [Reference #]	Country	Study Design	Data Source/Setting	Sample Size	Key Findings
Benfield et al., 2010 [98]	USA & Mexico	Randomized double-blind, placebo-controlled trial	Clinical assessment	274	Steroid withdrawal showed similar rejection rates and a higher 3-year graft survival compared with steroid maintenance.
Ruck et al., 2019 [63]	USA	National registry study	HLA typing	7497	Higher HLA mismatch was associated with a significantly increased risk of graft failure.
Hogan et al., 2023 [53]	International	Multicenter observational	iBox predictive model	706	The iBox score effectively predicted long-term graft failure risk.

## Data Availability

No new data were created or analyzed in this study. Data sharing is not applicable to this article.

## References

[1] Amaral S., Sayed B.A., Kutner N., Patzer R.E. (2016). Preemptive kidney transplantation is associated with survival benefits among pediatric patients with end-stage renal disease. Kidney Int..

[2] Lemoine C.P., Pozo M.E., Superina R.A. (2022). Overview of pediatric kidney transplantation. Semin. Pediatr. Surg..

[3] Fijo J., Sánchez-Moreno A. (2023). Life after a pediatric kidney transplant. Nefrologia (Engl. Ed.).

[4] Winterberg P.D., Garro R. (2019). Long-Term Outcomes of Kidney Transplantation in Children. Pediatr. Clin. N. Am..

[5] Roach J.P., Bock M.E., Goebel J. (2017). Pediatric kidney transplantation. Semin. Pediatr. Surg..

[6] Fernandez H.E., Foster B.J. (2022). Long-term care of the pediatric kidney transplant recipient. Clin. J. Am. Soc. Nephrol..

[7] Shapiro R., Sarwal M.M. (2010). Pediatric kidney transplantation. Pediatr. Clin. N. Am..

[8] Gorchs R., Stout M., Cohen B., Ju J., Brewer E., Galván N.T.N., Rana A. (2025). Risk factors for acute rejection in pediatric kidney transplantation. Transpl. Immunol..

[9] Zhang J., Pei J., Yu C., Luo J., Hong Y., Hua Y., Wei G. (2024). CCR7 and CD48 as Predicted Targets in Acute Rejection Related to M1 Macrophage after Pediatric Kidney Transplantation. J. Immunol. Res..

[10] Peticca B., Khalil I.Y., Robinson S.G., Prudencio T.M., Inlaw C., Majeethia H., Di Carlo A., Karhadkar S.S. (2025). Underappreciated Effects of Delayed Graft Function in Pediatric Kidney Transplantation. Pediatr. Transplant..

[11] Winnicki E., Copeland T.P., Kim A., Glenn Lecea E.M., McCulloch C.E., Ku E. (2025). Trends in Timing of Preemptive Kidney Transplantation and Association with Allograft and Survival Outcomes in Children. Clin. J. Am. Soc. Nephrol..

[12] Gu L., Gross A.C., Kizilbash S. (2025). Multidisciplinary approach to optimizing long-term outcomes in pediatric kidney transplant recipients: Multifaceted needs, risk assessment strategies, and potential interventions. Pediatr. Nephrol..

[13] Beyyumi E., Al Dhaheri W., Ahmad N., Zaman M.B., Khasawneh E.A. (2025). The Evolving Experience and Outcomes of Pediatric Kidney Transplant in Abu Dhabi, UAE (2010–2024). Int. J. Nephrol..

[14] Kindem I.A., Bjerre A., Hammarstrøm C., Naper C., Midtvedt K., Åsberg A. (2023). Kidney-transplanted Adolescents-Nonadherence and Graft Outcomes During the Transition Phase: A Nationwide Analysis, 2000–2020. Transplantation.

[15] Sreenivas A., Salgia E., Harish N., Raina R. (2024). Transition of care from pediatric to adult nephrology post-renal transplant: A review. Transl. Pediatr..

[16] Fadel F.I., Bazaraa H.M., Badawy H., Morsi H.A., Saadi G., Abdel Mawla M.A., Salem A.M., Abd Alazem E.A., Helmy R., Fathallah M.G. (2020). Pediatric kidney transplantation in Egypt: Results of 10-year single-center experience. Pediatr. Transplant..

[17] Shajari A., Ashrafi M.M., Shajari H., Derakhshan A. (2022). Graft and Patient Survival Rate among Iranian Pediatric Recipients of Kidney Transplantation: A Systematic Review and Meta-Analysis. Iran. J. Public Health.

[18] Wasik H.L., Pruette C.S., Ruebner R.L., McAdams-DeMarco M.A., Zhou S., Neu A.M., Segev D.L., Massie A.B. (2019). A donor risk index for graft loss in pediatric living donor kidney transplantation. Am. J. Transplant..

[19] Trnka P., McTaggart S.J., Francis A. (2018). The impact of donor/recipient age difference and HLA mismatch on graft outcome in pediatric kidney transplantation. Pediatr. Transplant..

[20] Yu J., Sheng X., Li Y., Sui M., Fu J., Zeng L., Li Y., Zhao W. (2025). Long-term outcomes and influencing factors following pediatric kidney transplantation: A single-center cohort study from China. Front. Pediatr..

[21] Koh L.J., Martz K., Blydt-Hansen T.D. (2019). Risk factors associated with allograft failure in pediatric kidney transplant recipients with focal segmental glomerulosclerosis. Pediatr. Transplant..

[22] Anand A., Malik T.H., Dunson J., McDonald M.F., Christmann C.R., Galvan N.T.N., O’Mahony C., Goss J.A., Srivaths P.R., Brewer E.D. (2021). Factors associated with long-term graft survival in pediatric kidney transplant recipients. Pediatr. Transplant..

[23] Hwang C.S., Kadakia Y., Sanchez-Vivaldi J.A., Patel M.S., Shah J.A., DeGregorio L., Desai D.M., Vagefi P.A., MacConmara M. (2023). Delayed graft function in pediatric living donor kidney transplantation. Pediatr. Transplant..

[24] Bulut I.K., Taner S., Keskinoglu A., Sezer T.O., Kabasakal C. (2019). Pediatric Kidney Transplantation in Patients With Urologic Anomalies. Transplant. Proc..

[25] Beetz O., Weigle C.A., Nogly R., Klempnauer J., Pape L., Richter N., Vondran F.W.R. (2021). Surgical complications in pediatric kidney transplantation-Incidence, risk factors, and effects on graft survival: A retrospective single-center study. Pediatr. Transplant..

[26] Zhu K.L., Feng Y.H., Hu M.Y., Cui K.X., Shang W.W., Liu L., Wang J.X., Wang Z.G., Zhang L.Y., Cheng F.M. (2022). Analysis of prognostic factors of pediatric kidney transplantation. Zhonghua Er Ke Za Zhi.

[27] De Santis Feltran L., Genzani C.P., Hamamoto F., Fonseca M.J.B.M., de Camargo M.F.C., de Oliveira N.L.G., de Freitas Amaral F.C., Baptista J.C., Koch Nogueira P.C. (2022). Encouraging outcomes of using a small-donor single graft in pediatric kidney transplantation. Pediatr. Nephrol..

[28] Sushkov A.I., Molchanova E.A., Kaabak M.M., Moysyuk Y.G. (2019). Pediatric kidney transplantation: Early and long-term outcomes following 1187 procedures. Khirurgiia.

[29] Cordinhã C., Rodrigues L., Carmo C., Gomes C., Macário F., Correia A.J., Alves R., Figueiredo A. (2019). Pediatric Kidney Transplantation: Experience of a Center Over 4 Decades. Transplant. Proc..

[30] Ratviset P., Panombualert S., Chathum K., Wisanuyotin S. (2024). Outcomes of pediatric deceased donor kidney transplant in northeast Thailand. Pediatr. Transplant..

[31] Nehus E.J., Liu C., Lu B., Macaluso M., Kim M.O. (2017). Graft survival of pediatric kidney transplant recipients selected for de novo steroid avoidance—A propensity score-matched study. Nephrol. Dial. Transplant..

[32] Benziane A., Boutennoune S. (2017). Pediatric Kidney Transplant: Experience at an Algerian Nephrology Department. Exp. Clin. Transplant..

[33] Boussetta A., Abida N., Jellouli M., Ziadi J., Gargah T. (2024). Delayed graft function in pediatric kidney transplant: Risk factors and outcomes. Exp. Clin. Transplant..

[34] Hogan J., Pietrement C., Sellier-Leclerc A.L., Louillet F., Salomon R., Macher M.A., Berard E., Couchoud C. (2017). Infection-related hospitalizations after kidney transplantation in children: Incidence, risk factors, and cost. Pediatr. Nephrol..

[35] Patel H., Rodig N., Agrawal N., Cardarelli F. (2021). Incidence and risk factors of kidney allograft loss due to BK nephropathy in the pediatric population: A retrospective analysis of the UNOS/OPTN database. Pediatr. Transplant..

[36] Gander R., Asensio M., Royo G.F., Molino J.A., García L., Madrid A., Ariceta G., Lopez M. (2018). Vascular thrombosis in pediatric kidney transplantation: Graft survival is possible with adequate management. J. Pediatr. Urol..

[37] Gargah T., Abidi K., Rajhi H., Ben Abdallah T., Chebil M., Lakhoua M.R. (2011). Vascular complications after pediatric kidney transplantation. Tunis. Med..

[38] Taher A., Zhu B., Ma S., Shun A., Durkan A.M. (2019). Intra-abdominal Complications After Pediatric Kidney Transplantation: Incidence and Risk Factors. Transplantation.

[39] Ladhani M., Lade S., Alexander S.I., Baur L.A., Clayton P.A., McDonald S., Craig J.C., Wong G. (2017). Obesity in pediatric kidney transplant recipients and the risks of acute rejection, graft loss and death. Pediatr. Nephrol..

[40] Ehren R., Habbig S., Krupka K., Ernst A., Bald M., König S., Murer L., Özçakar Z.B., Pohl M., Babenko N. (2022). Prevalence and potential relevance of hyperuricemia in pediatric kidney transplant recipients—A CERTAIN registry analysis. Pediatr. Transplant..

[41] Calvo-Herrera M.A., Serna-Campuzano A.M., Isaza-Lopez M.C., Villegas-Arbelaez E., Rojas-Rosas L.F., Serna-Higuita L.M., Ochoa-García C.L. (2024). “Effect of post-kidney transplant diabetes mellitus on long-term outcomes in a cohort of pediatric kidney transplant recipients from 2005 to 2022.” Survival analysis. BMJ Paediatr. Open.

[42] Martinez-Mier G., Mendez-Lopez M.T., Soto-Miranda E., Moreno-Ley P.I., Budar-Fernandez L.F., Rizo-Velazquez C.G., Vega-Rojano L. (2019). Acute rejection is a strong negative predictor of graft survival in living-donor pediatric renal transplant: 10-year follow-up in a single Mexican center. Exp. Clin. Transplant..

[43] Lim W.H., McDonald S.P., Kennedy S.E., Larkins N., Wong G. (2017). Association Between Slow and Delayed Graft Function With Graft Outcomes in Pediatric and Adolescent Deceased Donor Kidney Transplant Recipients. Transplantation.

[44] Yaffe H.C., Friedmann P., Kayler L.K. (2017). Very small pediatric donor kidney transplantation in pediatric recipients. Pediatr. Transplant..

[45] Lysakowski S., Druck Garcia C., Weisheimer Rohde R., Pascual Vitola S., Silva Pires F., Carla de Souza V., Enrico Ventura P., Kist R. (2024). Pediatric kidney transplantation: Outcomes with under and over 6-year-old donors. J. Pediatr..

[46] White M.H., Ross L., Gallo A., Parker W.F. (2024). Graft Survival of En Bloc Deceased Donor Kidneys Transplants Compared With Single Kidney Transplants. Transplantation.

[47] Selvathesan N., Han D.Y., Palmer S.C., Dickens A., Manley P., Prestidge C. (2024). Graft Loss in Pediatric and Young Adult Kidney Transplantation in New Zealand: Who Is at Greatest Risk and When?. Pediatr. Transplant..

[48] Jordan C.L., Taber D.J., Kyle M.O., Connelly J., Pilch N.W., Fleming J., Meadows H.B., Bratton C.F., Nadig S.N., McGillicuddy J.W. (2016). Incidence, risk factors, and outcomes of opportunistic infections in pediatric renal transplant recipients. Pediatr. Transplant..

[49] Møller D.L., Sørensen S.S., Wareham N.E., Rezahosseini O., Knudsen A.D., Knudsen J.D., Rasmussen A., Nielsen S.D. (2021). Bacterial and fungal bloodstream infections in pediatric liver and kidney transplant recipients. BMC Infect. Dis..

[50] Pérez-Bertólez S., Barrero R., Fijo J., Alonso V., Ojha D., Fernández-Hurtado M.Á., Martínez J., León E., García-Merino F. (2017). Outcomes of pediatric living donor kidney transplantation: A single-center experience. Pediatr. Transplant..

[51] Kaboré R., Ferrer L., Couchoud C., Hogan J., Cochat P., Dehoux L., Roussey-Kesler G., Novo R., Garaix F., Brochard K. (2021). Dynamic prediction models for graft failure in paediatric kidney transplantation. Nephrol. Dial. Transplant..

[52] Kostakis I.D., Chandak P., Assia-Zamora S., Gogalniceanu P., Loukopoulos I., Calder F., Stojanovic J., Kessaris N. (2023). Pediatric renal transplantation-A UNOS database analysis of donor-recipient size mismatch. Pediatr. Transplant..

[53] Hogan J., Divard G., Aubert O., Garro R., Boyer O., Donald Cooper L.A., Farris A.B., Fila M., Seifert M., Sellier-Leclerc A.L. (2023). Validation of a prediction system for risk of kidney allograft failure in pediatric kidney transplant recipients: An international observational study. Am. J. Transplant..

[54] Van Stralen K.J., Verrina E., Belingheri M., Dudley J., Dusek J., Grenda R., Macher M.A., Puretic Z., Rubic J., Rudaitis S. (2013). Impact of graft loss among kidney diseases with a high risk of post-transplant recurrence in the paediatric population. Nephrol. Dial. Transplant..

[55] Suh H. (2025). Identifying risk factors for graft failure due to chronic rejection <15 years post-transplant in pediatric kidney transplants using random forest machine-learning techniques. Pediatr. Transplant..

[56] Mincham C.M., Wong G., Teixeira-Pinto A., Kennedy S., Alexander S., Larkins N., Lim W.H. (2017). Induction Therapy, Rejection, and Graft Outcomes in Pediatric and Adolescent Kidney Transplant Recipients. Transplantation.

[57] Okada M., Kamei K., Matsuoka K., Ito S. (2018). Development of antibody mediated rejection shortly after acute cellular rejection in a pediatric kidney transplantation recipient. CEN. Case Rep..

[58] Borsum N., Verboom M., Ahlenstiel-Grunow T., Pape L. (2019). Cytokine Profiles in Children After Pediatric Kidney Transplantation With Acute Cellular Compared to Chronic Antibody-mediated Rejection and Stable Patients: A Pilot Study. Transplant. Direct.

[59] Serrano Pejenaute I., Lejarzegi Anakabe E., Madariaga L., Alonso Varela M., Santos-Diez Vázquez L., Herrero Goñi M. (2022). Secondary Immunosuppression in Pediatric Kidney Transplant Recipients. Exp. Clin. Transplant..

[60] Tönshoff B., Tedesco-Silva H., Ettenger R., Christian M., Bjerre A., Dello Strologo L., Marks S.D., Pape L., Veldandi U., Lopez P. (2021). Three-year outcomes from the CRADLE study in de novo pediatric kidney transplant recipients receiving everolimus with reduced tacrolimus and early steroid withdrawal. Am. J. Transplant..

[61] Tiwari A.K., Aggarwal G., Arora D., Bhardwaj G., Jain M., Bansal S.B., Sethi S.K. (2020). Immunoadsorption in ABO-incompatible kidney transplantation in adult and pediatric patients with follow-up on graft and patient survival: First series from India. Asian J. Transfus. Sci..

[62] Liu Q., Zhang H., Zhong M., Tan L., Hu S., Peng L., Xie X., Lan G. (2023). Excellent clinical outcomes of renal transplant from pediatric deceased donors with acute kidney injury. Eur. J. Med. Res..

[63] Ruck J.M., Jackson A.M., Massie A.B., Segev D.L., Desai N., Garonzik-Wang J. (2019). Temporal Changes in the Impact of HLA Mismatching Among Pediatric Kidney Transplant Recipients. Transplantation.

[64] Aoki Y., Hamasaki Y., Hashimoto J., Zaitsu A., Suda S., Itabashi Y., Muramatsu M., Kawamura T., Shishido S., Sakai K. (2024). Comparison of preemptive and non-preemptive kidney transplantation outcomes in children aged <6 years. Medicine.

[65] Troppmann C., Santhanakrishnan C., Fananapazir G., Troppmann K., Perez R. (2018). Pediatric en bloc kidney transplantation from very small (≤10 kg) donation after circulatory death (versus brain death) donors: Single-center matched-pair analysis of 130 transplants. Am. J. Transplant..

[66] Huang M., Wu S., Gao P., Zhou L., Fu Q., Wu C., Zhang H., Zheng Y., Su X., Wu W. (2025). The effect of low donor-to-recipient body weight ratio on graft survival after dual kidney transplantation from pediatric deceased donors. Ren. Fail..

[67] Park M.J., Baek H.S., Song J.Y., Choi N., Ahn Y.H., Kang H.G., Cho M.H. (2023). Effect of donor-recipient size mismatch on long-term graft survival in pediatric kidney transplantation: A multicenter cohort study. Kidney Res. Clin. Pract..

[68] Jackson K.R., Zhou S., Ruck J., Massie A.B., Holscher C., Kernodle A., Glorioso J., Motter J., Neu A., Desai N. (2019). Pediatric deceased donor kidney transplant outcomes under the Kidney Allocation System. Am. J. Transplant..

[69] Lentine K.L., Smith J.M., Lyden G.R., Miller J.M., Booker S.E., Dolan T.G., Temple K.R., Weiss S., Handarova D., Israni A.K. (2025). OPTN/SRTR 2023 Annual Data Report: Kidney. Am. J. Transplant..

[70] Bajaj S., Gershony S., Afshar K., Blydt-Hansen T.D. (2022). Clinical indicators of slow graft function and outcome after pediatric kidney transplantation. Pediatr. Transplant..

[71] Ashoor I.F., Dharnidharka V.R. (2019). Non-immunologic allograft loss in pediatric kidney transplant recipients. Pediatr. Nephrol..

[72] Tönshoff B., Ettenger R., Dello Strologo L., Marks S.D., Pape L., Tedesco-Silva H., Bjerre A., Christian M., Meier M., Martzloff E.D. (2019). Early conversion of pediatric kidney transplant patients to everolimus with reduced tacrolimus and steroid elimination: Results of a randomized trial. Am. J. Transplant..

[73] Seeman T., Myette R.L., Feber J. (2023). Hypertension in pediatric kidney transplantation. Pediatr. Transplant..

[74] Smith K.M., Windsperger A., Alanee S., Humar A., Kashtan C., Shukla A.R. (2010). Risk factors and treatment success for ureteral obstruction after pediatric renal transplantation. J. Urol..

[75] Hamasaki Y., Hashimoto J., Aoki Y., Kubota M., Muramatsu M., Kawamura T., Shishido S., Sakai K. (2022). Long-term social outcome after pediatric kidney transplantation: A single-center experience. Clin. Exp. Nephrol..

[76] Nunez M., Abbasi A., McEnhill M., Brennan J., Shappell T., Kinnier S., Winnicki E., Stock P. (2025). Long-term impact of immigration status on outcomes in pediatric kidney transplant recipients. Am. J. Transplant..

[77] Aoki Y., Satoh H., Hamasaki Y., Hamada R., Harada R., Hataya H., Ishikura K., Muramatsu M., Shishido S., Sakai K. (2022). Incidence of malignancy after pediatric kidney transplantation: A single-center experience over the past three decades in Japan. Clin. Exp. Nephrol..

[78] Zarauza Santoveña A., García Meseguer C., Martínez Mejía S., Alonso Melgar Á., Fernández Camblor C., Melgosa Hijosa M., Peña Carrión A., Espinosa Román L. (2015). BK virus infection in pediatric renal transplantation. Transplant. Proc..

[79] Parmaksiz G., Avci B., Noyan A., Baştürk B., Çalişkan K., Baskin E., Haberal M. (2024). Viral Infections in Pediatric Kidney Transplant Recipients: Effects on Graft Function, Risk Factors, and Patient Outcomes. Exp. Clin. Transplant..

[80] Canpolat N., Yıldırım Z.Y., Yıldız N., Taşdemir M., Göknar N., Evrengül H., Gülmez R., Aksu B., Dursun H., Özçelik G. (2022). COVID-19 in pediatric patients undergoing chronic dialysis and kidney transplantation. Eur. J. Pediatr..

[81] De Souza V.C., Rabilloud M., Cochat P., Wagner M.B., Garcia C.D., Ranchin B., Iwaz J., Selistre L., Dubourg L. (2017). Trajectories and Predictors of Allograft Dysfunction after Renal Transplantation in Children. Am. J. Nephrol..

[82] Gander R., Asensio M., Andrés Molino J., Fatou Royo G., Lopez-Gonzalez M., Perez V., López M., Ariceta G. (2022). Pediatric kidney retransplantation focused on surgical outcomes. J. Pediatr. Urol..

[83] Oomen L., de Wall L.L., Cornelissen E.A.M., Feitz W.F.J., Bootsma-Robroeks C.M.H.H.T. (2021). Prognostic factors on graft function in pediatric kidney recipients. Transplant. Proc..

[84] Coens F., Knops N., Tieken I., Vogelaar S., Bender A., Kim J.J., Krupka K., Pape L., Raes A., Tönshoff B. (2024). Time-varying determinants of graft failure in pediatric kidney transplantation in Europe. Clin. J. Am. Soc. Nephrol..

[85] Seifert M.E., Yanik M.V., Feig D.I., Hauptfeld-Dolejsek V., Mroczek-Musulman E.C., Kelly D.R., Rosenblum F., Mannon R.B. (2018). Subclinical inflammation phenotypes and long-term outcomes after pediatric kidney transplantation. Am. J. Transplant..

[86] Pearl M.H., Zhang Q., Palma Diaz M.F., Grotts J., Rossetti M., Elashoff D., Gjertson D.W., Weng P., Reed E.F., Tsai Chambers E. (2018). Presence of Angiotensin II Type 1 receptor antibodies is associated with increased risk of acute rejection and decline in graft function in pediatric kidney transplant recipients. Pediatr. Transplant..

[87] Pearl M.H., Chen L., Zuckerman J.E., Weng P.L., Chambers E.T., Zhang Q., Reed E.F. (2024). Non-HLA Antibodies to G Protein-coupled Receptors in Pediatric Kidney Transplant Recipients: Short- and Long-term Clinical Outcomes. Transplantation.

[88] Ettenger R., Chin H., Kesler K., Bridges N., Grimm P., Reed E.F., Sarwal M., Sibley R., Tsai E., Warshaw B. (2017). Relationship Among Viremia/Viral Infection, Alloimmunity, and Nutritional Parameters in the First Year After Pediatric Kidney Transplantation. Am. J. Transplant..

[89] Engen R.M., Keyser M., Jiang Z., Kizilbash S. (2024). Norovirus Management and Outcomes in a Multicenter Pediatric Kidney Transplant Population. Pediatr. Transplant..

[90] Serrano O.K., Gannon A., Olowofela A.S., Reddy A., Berglund D., Matas A.J. (2019). Long-term outcomes of pediatric kidney transplant recipients with a pretransplant malignancy. Pediatr. Transplant..

[91] Nada A., Jetton J.G. (2022). Pediatric Onco-Nephrology: Time to Spread the Word-Part II: Long-Term Kidney Outcomes in Survivors of Childhood Malignancy and Malignancy after Kidney Transplant. Pediatr. Nephrol..

[92] Order K.E., Rodig N.M. (2024). Pediatric Kidney Transplantation: Cancer and Cancer Risk. Semin. Nephrol..

[93] Kanzelmeyer N.K., Maecker-Kolhoff B., Zierhut H., Lerch C., Verboom M., Haffner D., Pape L. (2018). Graft outcomes following diagnosis of post-transplant lymphoproliferative disease in pediatric kidney recipients: A retrospective study. Transpl. Int..

[94] Pollack S., Plonsky M., Tibi R., Libinson-Zebegret I., Yakobov R., Eisenstein I., Magen D. (2025). Prevention of post-transplant lymphoproliferative disorder in pediatric kidney transplant recipients. Pediatr. Nephrol..

[95] Grenda R. (2022). Non-Hodgkin lymphoma after pediatric kidney transplantation. Pediatr. Nephrol..

[96] Kizilbash S., Rheault M., Matas A., Mauer M., Nevins T., Chavers B. (2024). More than four decades of graft survival in pediatric kidney transplant recipients. Pediatr. Nephrol..

[97] Chinnakotla S., Verghese P., Chavers B., Rheault M.N., Kirchner V., Dunn T., Kashtan C., Nevins T., Mauer M., Pruett T. (2017). Outcomes Risk Factors for Graft Loss: Lessons Learned from 1,056 Pediatric Kidney Transplants at the University of Minnesota. J. Am. Coll. Surg..

[98] Benfield M.R., Bartosh S., Ikle D., Warshaw B., Bridges N., Morrison Y., Harmon W. (2010). A randomized double-blind, placebo controlled trial of steroid withdrawal after pediatric renal transplantation. Am. J. Transplant..

